# Can overzealous reliance on evidence-based medicine overshadow good clinical judgement?

**DOI:** 10.4102/jcmsa.v2i1.30

**Published:** 2024-01-31

**Authors:** Leanne M. Sykes, Gerhard Grobler, Charles Bradfield

**Affiliations:** 1 Department of Prosthodontics, Faculty of Health, University of Pretoria, Pretoria, South Africa; 2 Department of Psychiatry, Faculty of Health, University of Pretoria, Pretoria, South Africa

**Keywords:** ethics, accountability, adverse events, evidence-based-medicine, clinical judgement

## Abstract

In the past, the doctor’s knowledge, clinical skills and judgement were the ‘main impetus for treatment decisions, therapy assessment and medical progress’, with the ‘emphasis being on protecting and restoring human well-being’. While the advent of evidence-based medicine (EBM) and the increased use of innovative technology have undoubtedly enhanced the practice of medicine and dentistry, overzealous reliance and application of these carries a risk of overshadowing the human aspects of clinical judgement and decision making. This paper uses a dental example to illustrate and caution practitioners against losing site of the patient in their quest to treat the condition. It is important for any practitioner who engages in radiology beyond conventional 2D imaging being liable to examine it in its entirety and to report on findings from the entire field of view. They may need theoretical and practical training to do this, as failure to detect and manage any pathology is considered negligence and is grounds for litigation. Alternatively, they can refer the patient to a trained radiologist if they would prefer an expert opinion. The area of dental radiography will be used for the following ethical and legal debate; however, the principles questioned could be applied to all specialist referrals. The ethical question relates to the issue of how to manage a patient where the clinicians based their treatment on the diagnosis and report given to them by an expert in some other related specialist medical field, but where the report was erroneous, resulting in an unforeseen adverse outcome. What makes the case more complex was the contradiction between the clinical judgement and the radiological findings, which only became evident at the time of surgery.

## Introduction

In the past, the doctor’s knowledge, clinical skills and judgement were the ‘main impetus for treatment decisions, therapy assessment and medical progress’, with the ‘emphasis being on protecting and restoring human well-being’.^1^ While the advent of evidence-based medicine (EBM) and the increased use of innovative technology have undoubtedly enhanced the practice of medicine and dentistry, overzealous reliance and application of these carries a risk of overshadowing the human aspects of clinical judgement and decision making.^1,2^ This paper uses a dental example to illustrate and caution practitioners against losing site of the patient in their quest to treat the condition. In a previous paper,^3^ the authors reported on the importance of ‘any practitioner who engages in radiology beyond conventional 2D imaging being liable to examine it in its entirety (not to only look at their area of interest) and to report on findings from the entire field of view’. In order to do this, they often need to undergo additional theoretical and practical training, as failure to detect and manage any pathology is considered negligence and is grounds for litigation.^4,5^ Alternatively, if a more complex diagnostic image and expert opinion is needed, the practitioner should rather ‘refer the patient to a trained radiologist who will be able to provide them and the patients with the most accurate and detailed report’.^3^ The area of dental radiography will once again be used as the basis for the following ethical debate; however, the principles questioned could be applied to all specialist referrals.

## Case scenario

A patient presented at the clinic having recently lost her maxillary central incisor tooth and requesting to have it restored with an implant-retained prosthesis. Both the restorative dentist and surgeon were not only concerned about bone levels in the area but also wanted to ensure that the fixture was placed in the best position and angulation for future aesthetic and functional occlusal needs. They referred the patient to a specialist radiographer to have a cone beam computer tomographic (CBCT) scan taken, which they then used to plan the size, site and angulation and thereafter to fabricate a surgical guide.

During the planning stages, they based their decisions on guidance from the expert’s report of where the bone level and density were the best and most suitable to ensure integration. In their opinion, this was not the most ideal position for their desired emergence site or angulation; however they went ahead and fabricated the guide and provisional restoration based on the technologically derived advice given. At the time of surgery, they discovered that there was adequate bone in the area where they had initially wanted to place the implant but had already prepared the guide and a provisional restoration that differed. They were torn between following their own clinical judgement and rather placing the implant in their preferred site or to go ahead and work according to the expert advice given. The former would entail refuting the expert’s opinion, abandoning the guide, placing implants ‘free-hand’ based on clinical experience and then making a new provisional restoration after the operation. Choosing the latter option that ‘disregarded their tacit knowledge’ and by association consideration of the patient’s best interests in favour of technologically driven solution could compromise the final aesthetic and functional needs.

The ethical question then is how to manage the situation where an adverse outcome of therapy was based on a less than accurate report given to the clinician by an expert in some other allied field (e.g. blood sample analysis, cultures, radiological images).

The clinicians acted to the best of their abilities following extensive consultation and expert advice. The experts themselves based their opinions on evidence obtained using the latest diagnostic imaging tools. However, computers do at times ‘morph’ images and fill in gaps when they encounter voids, based on pre-programmed algorithms and anatomical norms. When the radiographers studied the images they had no way of knowing if, where and to what extent that this may have occurred and, as such, unintentionally gave flawed advice. This resulted in a classic example of a situation where the ‘overzealous reliance and application of EBM’ and/or technology and the guidance emanating from it, replaced good clinical judgement.^2^ They could argue that their advice was intended to help in the clinical decision-making process and not to replace it.^2^ As such the onus was on the surgeon and prosthodontist to still evaluate the situation at the time of the operation and make the final judgement call based on ‘extrapolation for the evidence presented to them, but interpreted in light of the presenting conditions’, in other words to use ‘clinical judgement’.^2^

The question then arises as to who would be accountable for any possible future negative consequences? The case could be examined purely on legal grounds but in fact has far more ethical and psychological factors to consider in relation to both the clinicians and the patient.

## Legal guidelines

In terms of the law, one needs to assess whether this situation was an adverse event or negligence, malpractice or was it practitioners working outside their scope of practice, for each of the parties involved. The first is usually not grounds for litigation; however, the latter three may all lead to legal actions and carry varying penalties depending on their extent, severity and amount of damage caused.

Adverse event – An adverse event refers to a harmful or negative outcome that has occurred during or directly after a patient has received medical care. They may range in severity and type such as medication side effects, injury, psychological harm or trauma or death. These events generally happen by chance when a well-intentioned action turns out badly, because of factors outside the clinician’s control. Adverse events should be treated at the patient level and ultimately managed at the systemic level. When a patient experiences an adverse event, the healthcare provider should provide timely and appropriate treatment.^6^

Negligence – ‘Negligence’ refers to conduct, that is, how practitioners behave in particular circumstances. Medical negligence occurs when practitioners fail to exercise the standard of skill and care of reasonably competent practitioners in their branch of the profession.^7^ Thus, the more complicated the procedure, the greater the degree of skill and care required although the courts will take into account the resources available to the health care practitioner at the time.^7^ An error in diagnosis is not necessarily negligence, the test is whether a reasonable practitioner in the same branch of medical practice would have made a similar decision or error.^7^ However, a failure to inform patients when such incidents happen or warn them of the possible post-operative complications and consequences may constitute negligence.^7^

Malpractice – Medical malpractice refers to treatment provided by a health care professional that is deemed to be below the acceptable standard of care, and that results in serious personal injuries to the patient.^8^ It is broader than medical negligence because it also includes intentional acts or omissions.^7^ ‘Intention’ refers to doing something knowing full well it is illegal or unethical.^7^ Any intervention carries with it a risk of error or failure. Complications can and do occur. However, in determining negligence or malpractice, one must also consider the clinician’s intention and if their aims were beneficent. Questions to ask include: was the aim to provide a therapeutic benefit, to protect the patient, to prevent harm, to remove conditions that could lead to future harm and was the therapy aimed at promoting the patient’s best interests?^8^ Legally to determine malpractice there are four issues that need to be proven in the court of law: that the practitioner was licensed, as this establishes that he and/or she had a duty to his and/or her patients to be professional and take care of them; that they failed in their duties through mistakes and poor treatment; that the mistake caused an injury and that the injury resulted in damages.^7,8^ The damage may be of a physical nature but can also include other costs such as lost time and wages, psychological trauma, incapacity or the need for additional medical or dental expenses.^8^

Practising outside your scope of expertise – Scope of practice refers to the activities, procedures and interventions that a clinician is authorised and competent to perform within their profession. It is determined by their education, training, certification, acquired skills and country laws. It may also vary depending on the work setting and employer policies.^9^

Based on the above definitions and descriptions, all of the clinicians involved had acted professionally. They had carried out a thorough pre-treatment examination and made use of the required diagnostic aids as well as of more cutting-edge diagnostic imaging (e.g., CBCT). Some authors have criticised over-reliance on technology and artificial intelligence (AI) in decision-making processes for its ‘inability to provide an opportunity for the back-and-forth conversations that characterize physician’s personal interactions’.^10^ Yet in this case, the entire team had engaged in multidisciplinary discussions and used their combined expertise and knowledge to formulate what they deemed to be the most suitable treatment plan. They had then assembled all required armamentarium, materials and components prior to surgery and had each carried out the procedures within their respective scopes of practice to the best of their abilities. None of them acted with malicious intent and could not have foreseen the clinical picture that emerged. If judged using the ‘Reasonable Person Rule’, which states that ‘a person has acted negligently if they have departed from the conduct expected of a reasonably prudent person acting under similar circumstances’,^8^ their actions were not negligent. However, their main error may have been their over-reliance on EBM and/or technology. The risk of being obsessed with implementing AI-enabled advice is that clinicians become prone to cognitive biases wherein they anchor themselves to a particular diagnosis and provide treatment based on this advice. In so doing, they fail to balance and modify their actions based on experience, sound clinical judgement and patient-centred considerations.^10^ In this scenario, such blind adherence to the pre-determined plan prevented the clinicians from altering their strategy when they encountered the clinical difficulties. In so doing, they lost focus on the good of the patient, which ultimately resulted in the unfortunate adverse event.

Having established this, the next issue is to consider the ethical parameters of care with regard to informing the patient and possible follow-up actions to be taken.

## Ethical considerations

There are two ethical issues to consider in this case. The first relates to the fact that there was indeed a substantial investment of time and resources in the planning stages, and yet there was still a sub-optimal outcome. This talks to the issue of distributive justice. Can one justify spending so much valuable clinical time, expertise and costly diagnostic aids on a single patient rather than spreading the resources over a number of cases, especially as the outcome did not seem to justify the costs? Although it may be argued that this was not known or anticipated at the time, and all parties believed that their efforts were in the best interest of the patient.

A second consideration is that of the clinician’s duty of truth telling. This is particularly important if any form of remedial action needs to be taken or if complications surface at a later stage. The question is, how much information should the patient be given at the time of treatment, and how will it impact on the doctor–patient relationship if they are only told the full truth at a later date? This can be considered in terms of both the ‘reasonable practitioner standards’ and well as from a patient-centred view of the ‘reasonable person rule’. The latter considers how much information would a reasonable person expect to be told. The former can be measured using the so-called ‘Bolam test’ of what would a reasonable practitioner be expected to reveal? This test is based on the ‘premise of determining whether the actions of the clinician are in line with the actions of other medics who are in their position’, as such it can change according to the situation and their degree of experience.^11^

A further issue revolves around the sufficiency of information required to enable action-specific decision making. An overload of information to the patient, especially at a time when they be feeling vulnerable and reliant on their doctors for help, may be as detrimental to sound decision making as too little information. The context and process of providing such information could influence the trust, confidence and ultimate decision making. For example, the clinicians may discuss the situation with the patient as a collective team or at least in the presence of a nurse. The added colleagues could all take part in the interaction, vouch for the information given and witness the patient’s final decision and consent or refusal of the proposed remedial plans. For some patients, this united voice may be reassuring to them as they may feel they are gaining the best advice from a multidisciplinary team of experts. However, other patients may be overwhelmed by the encounter and could even experience a sense of being ‘ganged-up against’ or coerced into accepting the resolutions presented to them. The latter is a particular risk in patients with known or suspected mental instability, those who are vulnerable by virtue of illness, pain, emotional distress or educational, financial, cultural or language barriers.^12^

The clinician would then need to discuss what remedial actions could or should be taken at the time as well as in the short and long term should complications develop (and for how long would they be liable for repercussions of this treatment). In many instances, the manner in which the patient deals with immediate and later consequences of adverse events is determined by how honest the practitioner was with them from the outset. They should have an open and honest discussion with the patient as to whether there was anything that could be done to remedy or improve the clinical situation immediately. They should also consider possible future treatment needs and their associated biological, time, inconvenience and financial costs. This would include coming to an agreement of who would be expected to carry the monetary costs and for how long the clinician would be held accountable for this particular dental situation. Unfortunately, the ethical obligation to truth telling does not provide explicit guidelines on how much information needs to be shared with the patient. Perhaps then the answer may be to look for guidance from a psychological perspective.

This article followed all ethical standards for research without direct contact with human or animal subjects.

## Psychological factors and/or assessment

If the patient did not know anything about the clinical dilemma that arose, and how this impacted on the final outcome, they may be obliviously happy with their restoration. Provided there are no complications, they will be content in the knowledge that they received good and appropriate treatment. However, if there are complications, at the time or in the within a short period after completion, then the clinician as an ethical professional would be obligated to handle these. Now they would be faced with not knowing if it is too late to divulge what had occurred or to remain silent and just provide the best possible further treatment that they could.

If they only revealed all of the information to this patient at a later time, she may feel deceived and become angry and seek remediation. She may also lose trust in the doctors and the profession. No amount of future work will ever fully satisfy her as she will now always be cross checking and looking out for errors. Clinicians need to remember that the doctor–patient relationship is built on trust. Patients need to feel confident that their doctors will not only make educated, evidence-based clinical decisions, but if forced into situations where they have to make value judgement calls, that they will weigh these against patient-related factors such as affordability, autonomy and their patients’ best interests. The patient may also feel disrespected and abused. The act of concealing information cannot be justified as paternalism where the clinicians thought they were protecting the patient from mental anguish, as in this case, they all purposefully hid the truth.

On the other hand, if the clinicians go ahead and try to resolve or remedy the situation without telling her, are they guilty of deceit? Can this be justified as strong paternalism if they want to spare her the mental anguish of knowing that there were problems in her treatment? There is no way that the clinicians can predict the patient’s response to whichever of the options they chose. Personality factors as well as the individual’s psychological status at that point in time will influence their reactions. Furthermore, patients with certain personality disorders, such as those in cluster C of the DSM-5 (Diagnostic and Statistical Manual of Mental Disorders, Fifth Edition) classification, or those with anxiety and mood disorders may be particularly vulnerable to the opinion of others, especially those in a position of power, and as such be more vulnerable to undue influence.^13^

## Case appraisal

Hospitals and academic institutions have brought together collaboration between colleagues from complementary health care sectors. This, along with rapid advances in technology, havsdeveloped medicine into a science. There is now in any given field inputs and exchange of information from a wide web of sources, and treatment is often carried out by a multidisciplinary team. However, this new age of technology has also made it tempting for practitioners to lose sight of the fact that ‘medicine should still be a combination of practical science and artistry’ and not be drawn into focussing more on the condition than the patient’.^1,14^ To this end, they may become greatly immersed in striving to use the latest available diagnostic tools, tests and armamentarium and prescribe the evidently best treatment or medication, that they may lose sight of the need for clinical judgement. They then unthinkingly provide scientifically based treatment without taking into account the human factors of both their own cognition and the patient’s unique needs. The risk is that patients become seen and treated as ‘scientific cases’ rather than as individual people.^2^

At the same time, health care workers often have to make tough judgement calls when coming to a final decision. Yet, there are times when despite them having planned and executed their work according to the best ‘evidence-based’ practices and highest standards of care, they still fall prey to the unpredictable realities of the human body. They may themselves make mistakes or encounter unexpected and unforeseen adverse events over which they had no control. It is at times like this that their academic qualifications need to be replaced with the virtues of wisdom, prudence and humility, and they ‘realise that the personal dimension should always take precedence within the clinician-patient-family relationship’.^14^ They should admit their shortcomings openly and honestly and do everything in their power, within reason, to help correct or lessen the damages.

## Conclusion

The work of health care professionals is often mentally, physically and psychologically demanding. This is even more so for those working in settings where infrastructure, materials and resources are limited or where patients are socioeconomically compromised. At the opposite end of the spectrum, those working in a first-world setting are bombarded with a wide array of high-tech diagnostic aids and treatment alternatives. Deciding on which to use for each situation can be equally stressful, as failure to make use of available test and treatment modalities may be construed as negligence, while over-prescription of adjunct aids could be seen as over-servicing. It is also tempting to be so resolute about using the latest technology that they run the risk of confusing medical good with patient good. Evidence-based medicine and technology will only ‘provide real clinical benefits if the doctors using them are able to balance trust and scepticism’. They need to trust the latest technology or they will never try implementing it, but at the same time a healthy amount of scepticism will allow them to balance the risk by drawing on prior experience and good clinical judgement.^10^ Clinicians need to remember that the doctor-patient relationship is built on trust. Patients need to feel confident that their doctors will not only make educated, evidence-based clinical decisions, but if forced into situations where they have to make value judgement calls, that they will weigh these against patient-related factors such as affordability, autonomy and their patients’ best interests. Medicine is not a perfect science, and there are times when doctors face therapeutic uncertainties and need to acknowledge their limitations. In these situations, it becomes even more imperative that they are cognisant of their privileged position in the clinical decision-making process. Being a professional obligates them to assume a more holistic approach in which they evaluate and balance all possible actions to ensure that first and foremost they treat the patient and not the condition. Perhaps, they should also be reminded that the word hospital is derived from the word hospitality and therefore they should treat every patient according to its definition, which is to act with kindness, compassion, sympathy, helpfulness, benevolence and humanity.
