# Erratum: Can overzealous reliance on evidence-based medicine overshadow good clinical judgement?

**DOI:** 10.4102/jcmsa.v2i1.98

**Published:** 2024-07-18

**Authors:** Leanne M. Sykes, Gerhard Grobler, Charles Bradfield

**Affiliations:** 1 Department of Prosthodontics, Faculty of Health, University of Pretoria, Pretoria, South Africa; 2 Department of Psychiatry, Faculty of Health, University of Pretoria, Pretoria, South Africa

In the original published article, Sykes LM, Grobler G, Bradfield C. Can overzealous reliance on evidence-based medicine overshadow good clinical judgement? J Coll Med S Afr. 2024;2(1), a30. https://doi.org/10.4102/jcmsa.v2i1.30, there was an error in the year of the published date. The published date was given incorrectly as 31 January 2023. The correct published date should be 31 January 2024.

The publisher apologises for this error. The correction does not change the study’s findings, its significance or overall interpretation of its results or the scientific conclusions of the article in any way.

